# The Use of Ext. Belladonna in the Treatment of Obstinate Vomitings in Pregnant Women

**Published:** 1853-11

**Authors:** R. L. Scruggs

**Affiliations:** Louisiana


From the Southern Journal.

*The use of Ext. Belladonna in the Treatment of Obstinate Vomitings in Pregnant Women.* By R. L. Scruggs, M.D., of Louisiana.

It is not a little surprising that an article capable of promptly arresting so grave a disease as the obstinate, and even dangerous vomitings, which supervene in the course of pregnancy, should have been so entirely neglected or overlooked by the profession generally; particularly when it is remembered that M. Bretonneau, more than eight years ago, announced the important fact to the profession in Europe, and pointed out the circumstances under which it ought to be used, the manner of applying it, &c. In the many recent discussions and papers read upon the subject of the propriety of inducing premature labor for this disease, I am surprised to see no allusion made to this remedy whatever. Even in that excellent and unique work, published in 1851, by Charles D. Meigs, upon "woman and her diseases," no mention is made of it, notwithstanding he says that the affection is so untractable as to justify the induction of premature labor. M. Trousseau, in a clinical lecture, delivered at the Hospital Neckar, in January, 1848, thus alludes to M. Bretonneau's theory and practice in these cases.*

"Five years ago," remarked the professor, "a lady, pregnant for the first time, who, for six weeks, had vomited both liquids and solids, called in M. Bretonneau. He found the patient in a most alarming state—the affection progressed rapidly, and threatened to become inevitably fatal. This woman, when questioned, complained of sharp uterine pains. In a primipara, the fibres of the uterus are not broken in, if you will allow the expression, and not habituated to the process, and allow themselves to be distended with difficulty; and it is this which causes the pain. M. Bretonneau thought that the uterine pains were the cause of the other symptoms, and that if he succeeded in mastering them, he would overcome the sympathetic vomitings of the patient. Acting upon this idea, he covered the hypogastrum repeatedly with a mixture of belladonna; the vomitings ceased the same day, and recovery ensued. Sometime afterwards, he had occasion to observe another case, where the pains of the uterus did not exist; but he thought that even if the brain did not perceive the pains of the uterus, the ganglia might take note of them, and reaction occur. To modify these accidents, he believed it to be sufficient to prescribe the belladonna mixture, and was again gratified with complete success. The result of these and similar cases justifies him, he thinks, in laying down the following principle:

"Whenever, in a woman, pregnant for the first time, or many times, vomitings supervene during the course of gestation, frictions should be made upon the hypogastrium with a mixture of belladonna, and the vomitings will cease.

The professor then asks, "In what manner does the belladonna act? I confess it is impossible to determine. Can it be supposed that the fœtus, in being developed, painfully distends the fibres of the uterus; that the vomitings are sympathetic, like those which supervene in cystitis, for example? This is possible. Whether it be this or something else, it is upon this hypothesis that M. Bretonneau has employed his remedy. He has promulgated his

* Yandell's Letters from Paris.

theory, and has endeavored to confirm it by facts. The fœtus distends the uterus, the nervous ganglia take cognizance of it, and sympathetic vomitings are the consequence. This is the theory, which you may adopt or not, but which must be admitted to conform, with marvellous exactness to the therapeutical results."

I had but just seen these opinions of M. Bretonneau announced, when I had an opportunity of making a practical application of them. My first patient, however, presented other symptoms than those described by him, for the relief of which he prescribed the belladonna mixture with such confidence and success. The result in this instance was equally fortunate.

Called in consultation, July 14th, 1848, to Mrs. L. W. D., æ. 24. This lady had been married about two years, and had miscarried once during the time, at about the fourth month of utero-gestation. She had been attended for several days before I saw her, by an experienced and scientific physician, who, failing in his efforts to relieve her of a most distressing cough, solicited my assistance.

Pregnancy, at the time of my visit, had not been suspected ; but upon a more thorough examination of the case, assisted by the answers elicited from her by questions in reference to this condition, we satisfied ourselves of the existence of pregnancy. I immediately suggested to my colleague the theory of M. Bretonneau, and asked, if this theory be correct, might not the sympathetic irritation produced by the distended and fretted uterine fibres react as well upon the bronchial mucous membrane—thus producing cough—as upon the stomach ? He caught at the idea at once, and we directed equal parts of ext. belladonna and lard to be rubbed together, and frictions made with the mixture, every four hours until our return. The next morning we were much gratified to find that the cough had entirely disappeared, and the patient feeling, of course, greatly relieved. She got up in a short time, and continued to enjoy moderately good health until she removed to Memphis, when we lost sight of her, but understood she was taken ill some months afterwards, and after suffering for several days, was delivered of a dead fœtus, at about the seventh month of utero-gestation. Having repeatedly seen the vomiting return after having been arrested by the application of belladonna over the hypogastrium, and again arrested by the same means, as promptly as at first, I am inclined to think now, that had the belladonna been used again in her case she might have gone to her full term, and possibly borne a living child.

Since the occurrence of this case, I have had repeated opportunities of testing the virtue of this article in similar cases, and in no instance has it failed to relieve the patient. It may be proper to remark, however, that any complications that may be found to co-exist with this condition, such as gastritis, gastro-enteritis, constipation, &c., ought to be treated with their appropriate remedies; and when the vomiting has continued for a considerable time, I have usually applied cups, fomentations, &c., under the impression that the excessive vomiting itself had excited inflammation of the gastric mucous membrane. But this has probably been an unnecessary proceeding, since it would appear from the observations of some of the most distinguished physicians of Europe, that no such condition of the mucous membrane of the stomach has been found to exist in subjects examined after death from this disease. My own observations tend also to establish this fact. At least, I have repeatedly found that the most active means that could be used for the subduction of the supposed gastric inflammation, proved altogether unavailing until the belladonna was applied over the hypogastrium, when the vomiting has invariably ceased. Very recently I delivered a young married lady of a healthy female child, who, about the middle of December last, was taken with excessive vomiting, attended with such violent straining, that when I arrived, I found that the matters ejected from the stomach were streaked with blood. The stomach being also tender to the touch, I proposed at once the application of the cups. But no persuasion could induce her to allow scarifications, nor even dry cupping. Failing in this, I ordered a purgative enema, a stimulating foot bath, a mustard cataplasm over the stomach, and used a variety of antiemetical mixtures, but all to no purpose. I then applied a belladonna plaster over the hypogastrium, and very soon she was relieved of her nausea and vomiting, and had no return of it for eight or ten days, when the plaster was again resorted to, which relieved her as promptly as at first, and she had no return of it afterwards.

I have now under my charge a young married lady, pregnant about six months, who suffered for a considerable time before she applied to me for relief. The belladonna here, as usual, was prompt and effectual in stopping the vomiting. She made use of it once or twice afterwards upon feeling slight nausea, but she is now, and has been for several weeks, perfectly healthy and free from any trouble of that sort.

M. Dubois, while upon the subject of the "induction of abortion in the vomiting of pregnant women," during a recent discussion in the Academie de Medicine, "stated the results of his experience in relation to obstinate vomiting in pregnancy. In proof that this is oftener a more dangerous occurrence than is usually supposed, he stated that in the course of thirteen years he had met with twenty cases in which it had proved fatal. That obstinate vomiting is but the exaggeration of the natural sympathetic vomiting of pregnancy, and not due to any special lesion, is proved by the facts that at the autopsies nothing is found, and that when the process of gestation becomes arrested, whether spontaneously or artificially, the vomiting is ordinarily put an end to, although the woman may not be delivered until several days after, of a dead child, and may yet die of the effects of what she has undergone." (Amer. Journal of the Medical Sciences, January, 1853.)

The observations of Dubois, Bretonneau, Ems, Duclos, Trousseau, and others, seem to go to establish the fact, that, no matter how violent or continued the vomitings are in these cases, there is no real inflammation of the stomach produced by them, and consequently any anti-phlogistic measures resorted to in view of this condition of the stomach, would appear to be, to say the least of it, unnecessary. Notwithstanding my own observations tend to establish the same fact, yet I cannot recommend an entire neglect of such adjuvant measures as would naturally suggest themselves to the intelligent physician. The bowels, of course, ought to be attended to, and the cups, fomentations, poultices, &c., may, I think, be justifiably resorted to upon a mere suspicion of gastric inflammation, for the patient is but slightly inconvenienced by them, and they will certainly relieve any inflammation that may exist. But I must protest against the blister. It will do no good at the time, and prove a source of great annoyance to the patient afterwards.

I have also used the belladonna ointment in cases of painful menstruation, with apparent benefit but my exp rience with it, in the treatment of these latter cases, is too limited to justify me in recommending it with any great confidence.

I have used it recently in a very violent case of dismenorrhœa, and it appeared to assist in relieving the pain; but so many other measures were resorted to, at the time, for the relief of this young lady, that it is impossible to determine what part, if any, the belladonna acted in giving the relief. I think, however, it is worthy a still further trial in these cases.

In conclusion I would suggest that it may be applied much more conveniently, and with equal efficiency, to the hypogastrium, by spreading the extract, undiluted, upon soft leather, in the manner of usiug the emp. cantharides, than by the plan originally suggested, of rubbing it on with the hand. This plan has the advantage, first, of being more cleanly, and secondly, may be re-applied by the patient herself, at any time when pain or nausea is felt.

